# Bibliographical Notices

**Published:** 1839-10-05

**Authors:** 


### BIBLIOGRAPHICAL NOTICES.

*Observations on the Typhoid Fever of New England.* (Read at the Annual Meeting of the Massachusetts Medical Society, May 29, 1839.) By Enoch Hale, M. D., Attending Physician to the Massachusetts General Hospital. Boston: 1839. 8vo. pp. 78.

The memoir of Dr. Hale was read, as its title denotes, at the annual meeting of the Massachusetts Medical Society. It is descriptive of the typhoid fever of New England; that is, of the true typhoid fever, as distinguished from typhus, whether of the graver or milder variety. We are pleased to learn that the distinction between the two forms of fever which are most prevalent respectively in England and in France, seems to obtain favour, and is now almost generally adopted in America. Fevers so distinct in their character should always be separated in the description, not merely to distinguish them for the benefit of the practitioner who happens to meet with them, but for the advantage of those who do not witness the particular form of disease which is the subject of observation.

The typhoid fever of New England, as described by Dr. Hale, and by Dr. Jackson, (Report on Typhoid Fever,) is perfectly identical with the typhoid fever which is so minutely described by Dr. Louis and Dr. Chomel. As Dr. Hale remarks, no distinct description of the disease is given by the English writers, who confound the cases of this fever with those of typhus, and include the whole under one general term. But as the more frequent disease is certainly the typhus fever, the description of continued fever by the British should be regarded as applicable to the mild or the severer form of true typhus, in which there is no lesion of the follicles of the small intestine. The latter form of disease has not, at least of late years, appeared in New England, unless in a case or two of European origin.

Dr. Hale's object was to present a condensed view of the symptoms of typhoid fever to the members of the Medical Society of Massachusetts, and the lecture cannot, of course, contain many facts which are new to our readers. His details of symptoms are composed of a summary of those observed in the typhoid fever of the Massachusetts General Hospital. These will be found to correspond entirely with the symptoms of the typhoid fever of Paris, and of course establish the absolute identity of the diseases. At Boston no exception occurred as to the lesion of Peyer's glands; it existed to a greater or less degree in every one of thirty-three cases which were examined after death.

The mortality of the disease at the hospital was a fraction more than eleven per cent.; this is a good average for cases in which the treatment is begun after the disease had lasted several days. The mortality in typhoid fever differs very little at Boston from that observed at Paris in the best hospitals, and affords a proportionate loss under good treatment, which is extremely uniform. Typhus fever is far from observing the same regular ratio of mortality. At times it is scarcely a disease of any danger, at others it assumes the form of the most malignant epidemic. The same irregularity occurs in remittents, and distinguishes them from typhoid fever.

Dr. Hale makes a remark relative to the lesions of the glands of Peyer in children, which is founded on correct observation. That is, that these lesions are not rare in children affected with other diseases than typhoid fever. But the inflammation of the follicles is comparatively superficial, and does not extend to the sub-mucous cellular tissue, as in the true typhoid fever. As early as the year 1832 we were occupied with inquiries as to the relative frequency of typhoid fever in children and in adults, and as to the frequency of alterations of the follicles of the small intestine in other diseases than typhoid fever. Several diseases of children occasionally present this superficial inflammation and ulceration of these glands, especially measles, and less frequently scarlatina and variola. Cholera infantum is very commonly attended with this lesion, and within the last few days we detected it in a child dead of jaundice and remittent fever. The epidemic of measles, which occurred at Paris in 1832, offered numerous examples of disease of the glands of Peyer, but they were uniformly slightly altered, when compared with the lesions met with in typhoid fever.

This somewhat extended notice of a short memoir is not in accordance with our usual custom; but we are so much gratified at researches relative to diseases which are still not universally understood, that we have pointed out at some length the more prominent facts of Dr. Hale's memoir. In one respect he is, perhaps, in error. He states that the lesion of the follicles of the small intestine is regarded by many in France, especially the pupils of Louis, as the chief cause of all the phenomena. This is certainly an error; the general belief, as far as we know, is, that the disorder of the glands causes a set of symptoms indicative of the lesion; but that the numerous symptoms of the fever cannot be supposed to depend upon a lesion, which, although very regular in its appearance, is by no means in all cases of sufficient extent to modify the course of the disease.

---

Plates on the Arteries, with References. By Paul B. Goddard, M. D., Demonstrator of Anatomy in the University of Pennsylvania, &c. &c. Philadelphia, J. G. Auner, 1839.

This is an exceedingly well executed work, one of the very best that has issued from the American press. It contains twelve capital plates, several of them original, and all of them differing in some modifications from previous works. The author informs us that he has followed the descriptions given in Horner's Special Anatomy, "the accuracy and faithfulness of which his own experience fully verifies." The combined authority of two such anatomists is of great weight. Dr. Goddard has omitted the surgical anatomy of the arteries, as unnecessarily complicating the views, and has confined himself to the representation of the vessels in their relations to the bones, ligaments, and muscles. These are presented with great clearness, and in a style most creditable to the author, and to the artists whom he has employed. The work cannot fail to become a text-book of the student of anatomy, and deserves a place, as a reference, in the library of the practitioner.

